# Predictors of Recent Incidence Trends in Sexually Transmitted Infections: A Systematic Review Protocol

**DOI:** 10.12688/hrbopenres.14222.1

**Published:** 2025-08-11

**Authors:** Santiago Garcia Guerrero, Robbie Lawlor, Ashling Bourke, John P. Gilmore, Caroline Kelleher, Maria Lohan, Nicola O'Connell, Kate O'Donnell, Rikke Siersbaek, Giovanni Villa, Chris Noone

**Affiliations:** 1 School of Psychology, University of Galway, Galway, County Galway, Ireland; 2 School of Human Development, Dublin City University, Dublin, Leinster, Ireland; 3 School of Nursing Midwifery and Health Systems, University College Dublin, Dublin, Leinster, Ireland; 4 School Of Population Health, Department of Health Psychology, Royal College of Surgeons in Ireland, Dublin, Leinster, Ireland; 5 School of Nursing and Midwifery, Queen's University Belfast, Belfast, Northern Ireland, UK; 6 Institute for Advanced Study, Hitotsubashi University, Kunitachi, Tokyo, Japan; 7 Sexual Health Programme, Health Service Executive, County Dublin, County Dublin, Ireland; 8 Health Protection Surveillance Centre, Dublin, Co. Dublin, Ireland; 9 Trinity College Dublin School of Medicine, Dublin, Leinster, Ireland; 10 Department of Genitourinary Medicine and Infectious Diseases, St James's Hospital, Dublin, Leinster, Ireland

**Keywords:** sexually transmitted infections; gonorrhoea; chlamydia; syphilis; HIV

## Abstract

**Introduction:**

Sexually transmitted infections (STIs) are a major global health concern, with millions of new cases occurring annually, particularly among young adults. These infections can lead to serious health complications, including infertility and increased risk of HIV, and are compounded by social stigma and mental health challenges. There have been significant global increases in STI diagnoses in recent years. The objective of this systematic review is to synthesise evidence on the predictors of trends in gonorrhoea, chlamydia, syphilis, and HIV over the last ten years. We aim to provide insight into the multifaceted drivers of the recent increasing STI diagnosis rates.

**Methods:**

We have developed a comprehensive search strategy that includes searching for relevant published literature and grey literature. We will include studies that contain evidence of longitudinal associations between changes in the incidence of diagnoses of four targeted STIs (i.e., gonorrhoea, chlamydia, syphilis, and HIV) during the last ten years. In addition, we will explore changes in sociodemographic and behavioural variables during the same time among representative samples of national populations. We will conduct a narrative analysis of the included studies.

**Discussion:**

The proposed synthesis plan is part of a larger research project that has been designed in response to the priorities of sexual health policymakers in Ireland. It will provide useful information regarding recent international trends in diagnoses of gonorrhoea, chlamydia, syphilis, and HIV, which will inform further efforts to understand the recent increases in STI diagnoses in Ireland. We acknowledge that it will be limited by publication bias, the biases affecting the included studies, a potential lack of data on important sub-populations, and restrictions related to testing availability across countries. Ultimately, trends in STI diagnoses are best understood through the design of comprehensive behavioural surveillance systems, which this review may usefully inform.

Reducing sexually transmitted infections (henceforth STIs) is an international public health priority. Globally, over 30 known pathogens are transmissible during sexual contact ( Torrone *et al.*, 2021), with STIs such as gonorrhoea, syphilis, chlamydia, trichomoniasis, hepatitis B, human papillomavirus (HPV), herpes simplex, and human immunodeficiency virus (HIV) being the most common contributors to the global disease burden ( Torrone *et al.*, 2021). According to the WHO (2024), more than one million curable STIs are acquired every day worldwide in people aged 15–49 years old, the majority of which are asymptomatic. Monitoring global trends of STIs is vital as these pathogens have a direct impact on physical, sexual, and reproductive health through pelvic inflammatory disease, infertility, cancers, and pregnancy complications, and can increase the risk of HIV ( WHO, 2024). Furthermore, the impact of STIs extends beyond physical health. Conditions such as HIV are closely linked with mental health challenges, including elevated rates of depression, anxiety, and post-traumatic stress disorder, often exacerbated by persistent social stigma and discrimination ( Ayano *et al.*, 2021; Brandt *et al.*, 2018; Tang *et al.*, 2020). Given the social complexity, the health challenges, and the economic burden associated with STIs, it is critical to monitor how global trends in STI diagnosis change and what drives these changes. Recent evidence suggests that STI diagnoses have been increasing worldwide over the past several years ( Elendu *et al.*, 2024; Mitjà *et al.*, 2023; Ong *et al.*, 2025; Sinka, 2024; WHO, 2024). The reasons for this increase in STIs are multifarious and multi-causal, and synthesising evidence from longitudinal studies can provide insights into how certain factors contribute, relate, and evolve over time, providing greater level of certainty than insights from cross-sectional studies.

The complexity of researching global trends in STIs is underscored by recent studies. In sub-Saharan Africa, for example, although global HIV incidence is in decline, the region still accounts for around half of all new infections. Women at high risk for HIV also face elevated rates of other STIs, as well as unintended pregnancies ( Cowan *et al.*, 2025). Although biomedical HIV prevention tools such as condoms, pre-exposure prophylaxis (PrEP), post-exposure prophylaxis (PEP) and rapid diagnostic tests have advanced, Cowan *et al.* (2025) argue that greater efforts are required to address the broader STI landscape, such as counteracting the effects of gender inequality ( Adeyemi, 2011; Nankinga *et al.*, 2016). To further highlight these complexities, targeted interventions addressing health disparities among marginalised groups such as ethnic minorities and individuals affected by racism, homophobia, transphobia, xenophobia, and socioeconomic inequities, have demonstrated effectiveness in reducing STI prevalence ( Bartholomew *et al.*, 2011; Lane *et al.*, 2004; Valleroy *et al.*, 2023). While understanding interventions for reducing STIs is important, it is also crucial to examine the changing dynamics of STI transmission to ensure policies remain responsive and up to date. 

Sexually transmitted infections are frequently framed as the result of individual behaviour such as condomless sex, having sex with multiple partners, or infrequent testing ( Chialepeh & Sathivasusuman, 2015; Marfatia *et al.*, 2015; Veličko *et al.*, 2016). However, this perspective neglects the deep societal and structural forces that shape exposure and vulnerability to STIs and access to care. Crowley *et al.* (2021) argue that STI transmission patterns are best understood through a broad sexual health paradigm that situates behaviour within a landscape of racism, stigma, healthcare access, and evolving technological and social norms. Their US-based research highlights that structural determinants, not just individual decisions, are central to understanding what drives STI epidemics.

In agreement with the above, Elendu *et al.*’s (2024) narrative review highlights many interconnected categories driving the increasing rates of STIs, namely structural factors, individual and network behaviours, biological challenges, and health system limitations. Globally, poverty, stigma, gender inequality, and the criminalisation of minority groups, such as the trans community, sex workers and gay and bi men create conditions where prevention tools are inaccessible, and diagnosis and care are often delayed ( Kavanagh *et al.*, 2021). STIs are mostly prevalent where condoms, testing services, treatments and/or vaccines are not available, or where people fear legal or social repercussions for seeking care ( Sinka, 2024). Weak sexual education programmes, growing misinformation networks ( Dangerfield, 2025; Elendu *et al.*, 2024), and the rise of hookup or dating apps ( Orellana *et al.*, 2024) reshaping access to sexual networks and behaviours have been identified as drivers of STI transmissions. Other factors such as substance use, including alcohol, methamphetamine, GHB (gamma-hydroxybutyrate), cocaine, and opioids, compound increased exposure to STIs by reducing condom use, increasing number of sexual partners and often delaying healthcare seeking ( Crowley *et al.*, 2021; Glynn *et al.*, 2018).

Adding complexity to the phenomenon, there are factors inherent to the coevolutionary host relationships of these pathogens ( Pimenoff *et al.*, 2018), reflected in changes in the genomic epidemiology of STIs, which collectively contribute to the development of drug-resistant strains ( Stary, 2020), variants with higher likelihood of reinfection (e.g., Alfsnes *et al.*, 2020; Brunham *et al.*, 1996), and initially undetected spread ( Mitjà *et al.*, 2023). Infrastructurally, shifts in consultations from face-to-face services to online services—where available—have been accompanied by an increase of self-testing and uptake of STI services, but the proportion of positive results may not necessarily be higher than face-to-face diagnoses for “high risk” individuals ( Turner *et al.*, 2019). Moreover, sociopolitical variables inevitably affect the epidemiological landscape of STIs, as lack of funded access to health services and interventions impact the overall reduction of STI diagnoses ( Leichliter *et al.*, 2017); an effect that, like interventions, may take several years to become apparent at the population level ( Hughes & Field, 2015). Together, these intersecting drivers highlight the complexity of STI transmission and reinforce the need for coordinated, systems and rights-based approach to the global STI approach.

Ong *et al.* (2025) build on the aforementioned evidence arguing that behaviour-change campaigns alone will not reverse STI trends. Alongside Elendu *et al.* (2024) and Crowley *et al.* (2021), they emphasise that equity must be central to STI responses as social and structural inequities are major drivers of STI rates. For example, “at home testing” initiatives presume stable housing, and digital illiteracy may risk excluding underserved communities ( Ong *et al.*, 2025). Without investment in healthcare systems, policy reform, and meaningful community involvement in service design, even the most advanced sexual health strategies risk reproducing the same disparities they aim to address ( Crowley *et al.*, 2021; Elendu *et al.*, 2024; NICE, 2022; Ong *et al.*, 2025). Ultimately, to provide effective STI prevention methods, efforts must go beyond encouraging “safe” behaviours. This requires confronting the structural and systemic conditions that shape behaviours and equitable access to services. Recognising and addressing the underlying drivers of STIs are essential for developing meaningful, just and impactful public health responses.

This systematic review synthesises recent literature on the predictors of international STI trends, with a specific focus on four common STIs: chlamydia, gonorrhoea, HIV and syphilis. These targeted STIs have been initially identified as the priority based on both availability (e.g., part of Ireland’s free-to-use “sexual health 24 hours a day; SH:24” self-testing service) and most costly to Ireland’s Health Service Executive (HSE) healthcare system.

By examining global epidemiological data, this review aims to provide a nuanced understanding of the multifaceted drivers of increasing STI rates. In doing so, it seeks to inform public health strategies and highlight the need for culturally competent, structurally informed interventions that address the complex and interconnected challenges that drive STIs.

Specifically, this review will address the following question: What are the predictors of incidence trends in diagnoses of gonorrhoea, chlamydia, syphilis, and HIV over the last ten years?

## Methods

### Study reporting and registration

We adhered to the Preferred Reporting Items for Systematic review and Meta-Analysis Protocols (PRISMA-P; Moher *et al.*, 2015) checklist in developing this protocol. We provide the PRISMA-P checklist in an Open Science Framework project ( https://osf.io/48m9p/). We also preregistered this study using the Open Science Framework (see https://osf.io/hdb2e).

### Eligibility criteria

We developed the inclusion criteria for the systematic review by applying the Population, Exposure, and Outcome (PEO) framework to our research question as we were interested in studies of the general adult population (P), sociodemographic and sexual behaviour data (E), and incidence trends of diagnoses of gonorrhoea, chlamydia, syphilis, and HIV over time (O). This informed both our search strategy and screening strategy. The inclusion criteria specifies that the studies:

- be written in English,
- report predictors of our targeted STI diagnoses (i.e., gonorrhoea, chlamydia, syphilis, HIV),
- report longitudinal population data on incidence changes over time for the targeted STI outcome variables (e.g., trend analysis via regression, time series, etc.),
- report data collected between 2015 (inclusive) and 2025, and
- report data from nationally representative samples (i.e., random sampling)

Sex and gender differences will be taken into consideration as reported in the sampled research (these sex/gender categories will be kept as per original sources).

The type of studies that we expect to sample include quantitative observational studies, surveillance data with statistical models, ecological analyses, time-series analyses.

### Information sources and search strategy

The key search concepts were based on the outcome variables of interest, and the methodological focus of the review as follows:

- Outcome: gonorrhoea ( *Neisseria gonorrhoeae*), chlamydia ( *Chlamydia trachomatis*), syphilis ( *Treponema pallidum*), HIV ( *human immunodeficiency virus*), sexually transmitted infections (STIs), sexually transmitted diseases (STDs).
- Study types: trend, longitudinal.

We generated a corresponding search string for each database (applying appropriate truncation and wildcards, and mesh/tree terms), following Bramer *et al.*’s ( 2018) guidelines on search standardisation (see https://osf.io/48m9p/).

These guidelines suggest that search strategies should be developed by:

1. Determining a clear and focused question
2. Describing the articles that can answer the question
3. Deciding which key concepts address the different elements of the question
4. Deciding which elements should be used for the best results
5. Choosing an appropriate database and interface to start with
6. Documenting the search process in a text document
7. Identifying appropriate index terms in the thesaurus of the first database
8. Identifying synonyms in the thesaurus
9. Adding variations in search terms
10. Using database-appropriate syntax, with parentheses, Boolean operators, and field codes
11. Optimising the search
12. Evaluating the initial results
13. Checking for errors
14. Translating to other databases
15. Testing and reiterating

The final search strategy was reached by testing all potential key concepts related to the focus of the present review. We systematically and progressively eliminated concepts that were considered redundant or were encompassed by other concepts, until the results obtained from the search strategy were both sensitive (i.e. picking up relevant papers) and reasonably specific in terms of the number of records being filtered.

This search strategy includes both peer-reviewed papers in academic journals as well as grey literature (e.g., conference papers, abstracts, reports, etc., containing sufficient information to fulfil the inclusion criteria). We will search relevant published literature via PubMed, Embase, CINAHL, psychINFO, and Scopus, and relevant grey literature via CINAHL, PsycINFO, Scopus, Google Scholar Medrxiv, and OSF Preprints. Grey literature from reports governmental bodies (e.g., WHO) will also be considered.

Citation mining (forward and backward) will be conducted on the final sample of selected papers (and on “benchmark articles”) using the Citation Chaser tool, R package “citationchaser” ( Haddaway *et al.*, 2022).

Periodic notifications for papers based on the saved search strategy will be reviewed for additional eligible publications up to the synthesis phase.

### Data management

All citations identified will be imported into the Zotero reference management software.

### Selection process

Three researchers will carry out the screening process using Covidence as outlined below.

### Title and abstract screening

Three reviewers will independently double screen the first 10–20% of the titles and abstracts and full texts against the exclusion criteria. Studies yielding divergent decisions between the reviewers will be assessed and discussed until consensus is reached (thereby calibrating the assessment approach).

Once inter-reviewer agreement is established, the remainder of the records will be distributed equally among two reviewers to be single screened independently. Any records for which a particular reviewer is unsure will be re-assessed in conjunction with the other reviewers.

### Full text screening

At full text screening, all texts will be double screened. The numbers of studies included and excluded, with reasons for exclusion, will be presented in a PRISMA flow diagram.

### Exclusion criteria

Papers will be excluded if they meet any of the following criteria:

- Off-topic: Our search may detect papers that are related to our outcome variable (incidence of specific diagnoses and STIs in general), but whose focus is not on epidemiological trends. For example, there might be studies targeting educational programmes or laboratory bacteriological studies, or there could be theoretical/discursive papers.
- English: While many abstracts might be in English (hence picked up by literature searches), we will only include studies whose content and data (i.e., full text) are written in English. An exception might be made for high quality peer-reviewed papers published in Spanish and Italian (matching the researchers’ spoken languages), so long as these are selected by the English-based searches.
- Untargeted STIs: Studies that focused on STIs other than gonorrhoea, chlamydia, syphilis, HIV (e.g., Lymphogranuloma venereum, hepatitis B virus, monkeypox, etc.).
- Non-trend data: Studies whose data does not correspond to changes of the outcome variable over time (viz., longitudinal), or at least two points in time—spaced apart by a significant/sensitive period (e.g., ≥ 1 year) for the phenomenon of interest; as well as studies whose data do not include predictors of our targeted STI outcomes. Purely descriptive (with no predictor analysis) or cross-sectional studies without time dimension will not be considered.
- Data > 10 years: Studies reporting data beyond our specified time period of 2015–2025.
- Non-representative sample: Sample is not nationally representative.

The foregoing criteria were pilot-tested with a random sample of papers by two reviewers so that the criteria could be clarified.

### Data collection process

The extraction of relevant data will be conducted in the following stages:

1. Piloting
1.1 *Test/design*: For this stage, two papers from the sample will be randomly selected and their data will be extracted using an extraction form. This will be done by reviewer one, and a second reviewer will repeat the extraction with one of these papers. Sources of digression between the two reviewers or ambiguity will be discussed, and the extraction form will be modified accordingly to improve its recording consistency.
1.2 *Training*: Two reviewers will select a paper—not selected for the previous step—and go through the extraction procedure again.
1.3 *Consistency verification*: The consistency in the data extraction is assessed. If there is considerable inconsistency, the process is repeated from the test/design stage.
2. Extraction
2.1 *Single extraction*: The first reviewer will extract data from all the sampled records.
2.2 *Reliability verification*: After the first reviewer has extracted data from all the sampled records, the second reviewer will repeat this process with 10% of the records. This extracted data will then be compared against the data extracted by the first reviewer to judge the consistency between the two extractions/reviewers.
2.3 *Consistency verification*: Corroboration of matching extraction validates and terminates the procedure.

A spreadsheet template containing extraction comments will be developed during the piloting stage, based on the data items indicated in the next section.

### Data items

Since we will be producing a narrative synthesis, we will document:

1. Population and setting;
2. Exposures of interest; predictors and variables associated with changes over time of STIs incidence. That is, sociodemographics, behaviours (e.g., condom use), testing rates, policy changes, socioeconomic factors, etc.;
3. STI outcomes; numerical data used for trend analyses over predictors of the targeted STIs such as on the population size (use as denominator for the studies’ calculations), frequency counts of new cases, percentage of increase, period(s) of prevalence, years surveyed, incidence changes, statistical models linking predictors to temporal trends in STI incidence, etc.;
4. Predictors analysed, significant effects, and their direction;
5. Factors reported as potential moderators and their estimations of associations or effect sizes (e.g., mean differences, χ2 differences in proportions);
6. Descriptions of the methods used such as the included studies’ designs, sample sizes, data collection intervals, blinding procedures, etc.;
7. Risk of bias indicators; and
8. Metadata such as authors and year of publication.

Where necessary, we will contact authors regarding missing data.

### Data management and sharing

All data and materials (e.g., RIS lists, CSV spreadsheets, R-scripts, supplementary documentation, etc.) from the project will be made available in due time (after publication of the respective paper) via the projects’ OSF repository (see https://osf.io/48m9p). We will aim to abide by FAIR (Findable, Accessible, Interoperable, and Reusable) open data recommendations.

### Outcomes and prioritisation

We will attempt to extract—reported—data linking predictors of incidence on the targeted STIs. This could include information on the population characteristics (e.g., PROGRESS stratifying factors), age, sexuality/sexual identity, behavioural factors (e.g., sex practices, contraception method, number of sex partners, etc.), and moderating factors (e.g., social affiliations, previous history of STIs, comorbidity/co-infections, substance use, etc.).

Likewise, we will extract data on frequency counts of STI cases recorded, percentage change, rate change, proportions of occurrences. If the available trend data allows for aggregation across studies, such data will be graphed. Customary epidemiological trend calculations will be considered. For example, incidence rate (per 100,000 population) will be calculated by dividing the number of new cases of STIs by the population size to date. Additionally, percentage of rate change can be a useful approach to convey trends—in particular, if only incidence figures are reported in the literature—and it will be calculated by subtracting the most recent count of cases from the previous count, and dividing such a number by the previous count.

### Risk of bias in individual studies

Given the study design of interest, we compiled a quality appraisal form (see https://osf.io/48m9p/) based on the National Health, Lung, and Blood Institude ( NHLBI, 2021) Quality Assessment Tool for Observational Cohort and Cross-Sectional Studies, and the Joanna Briggs Institute (JBI): Critical Appraisal Tools for Prevalence Studies ( Munn *et al.*, 2015).

### Data synthesis

It is unlikely that the studies included in this systematic review will be similar enough for meta-analysis to be appropriate. The present systematic review will therefore synthesise the information from the sample (with due attention to their biases, strengths, and limitations) via narrative synthesis. We will follow the Synthesis Without Meta-analysis (SWiM) reporting guidelines ( Campbell *et al.*, 2020).

We took inspiration from the ESRC Methods Programme’s Guidance on the Conduct of Narrative Synthesis in Systematic Reviews ( Popay *et al.*, 2006) in developing our synthesis plan. Specifically, we will conduct narrative synthesis by conducting a thematic analysis of how the results of the included studies are described and interpreted. If possible (subject to data availability and homogeneity), we will produce tabular summaries of factors associated with trends in the target STIs. Likewise, if consistency in the reported data allows it, summaries will be organised by yearly increments dating back from 2015 to 2025. Otherwise, broader time periods will be used (e.g., a two-point report might be adopted; as in the earliest, and last year reported by all studies) or some other way of conveying the averaged data trends (e.g., thematic categorization based on increases of varying magnitudes of incidence increase/decrease). In addition, any differences based on sex/gender will be disaggregated and discussed separately.

### Meta-bias(es)

Given the likely heterogenous nature of the studies that will be included in this review, and our use of narrative analysis, it is not possible to quantify the presence of publication bias or other forms of meta-bias. Where possible, we will try to identify selective outcome reporting by comparing the included studies to their protocols or preregistrations.

### Confidence in cumulative evidence

We considered the application of GRADE to evaluate our confidence in evidence synthesised in this review, but it was deemed unfeasible due to the likely wide range of effects that will be included, and the lack of a focus on evidence regarding specific interventions.

### Limitations

There are two major limitations with the proposed review. First, it is likely that there will be significant heterogeneity in the factors examined in the literature in relation to STI prevalence rates. This will make it difficult to disentangle differences in results from methodological differences. Secondly, it is expected that many factors that affect the transmission of STIs will not have been measured in the studies that are included in this review. We will take these limitations into account in the interpretation of the review’s findings.

## Data Availability

None. Open Science Framework: Systematic Review | PREDICTORS OF RECENT INCIDENCE TRENDS IN SEXUALLY TRANSMITTED INFECTIONS. https://doi.org/10.17605/OSF.IO/48M9P ( Garcia-Guerrero *et al.*, 2025) This project contains the following extended data: Search strategy. (Full search strategy for 5 databases.) Quality Assessment Tool. (Quality appraisal form compiled and adapted based on: the NHLBI Quality Assessment Tool for Observational Cohort and Cross-Sectional Studies, and the Joanna Briggs Institute (JBI): Critical Appraisal Tools for Prevalence Studies ( Munn *et al.*, 2015)) Data Extraction Tool. (Spreadsheet for use during data extraction) Open Science Framework: Appendix 1 - PRISMA-P checklist for 'Predictors of Recent Incidence Trends in Sexually Transmitted Infections: A Systematic Review Protocol’. https://doi.org/10.17605/OSF.IO/48M9P ( Garcia-Guerrero *et al.*, 2025). Data are available under the terms of the Creative Commons Attribution 4.0 International license (CC-BY 4.0).
