# Supplementary material for: Predictors of Recent Incidence Trends in Sexually Transmitted Infections: A Systematic Review Protocol
Source: HRB Open Res. 2026 Jan 8;8:88. Originally published 2025 Aug 11. [Version 2] doi: 10.12688/hrbopenres.14222.2 (PMC12859410; doi:10.12688/hrbopenres.14222.2)
Supplement: Peer review history

# Reviewer report: Reviewer response for version 2

**DOI:** 10.21956/hrbopenres.15748.r52745

**Published:** 2026-01-30

**Authors:** Chantal den Daas

**Affiliations:** 1 University of Aberdeen, Aberdeen, UK

I think the response to my queries was detailed and realistic. Authors have addressed all concerns I had.

Is the study design appropriate for the research question?

Yes

Is the rationale for, and objectives of, the study clearly described?

Yes

Are sufficient details of the methods provided to allow replication by others?

Yes

Are the datasets clearly presented in a useable and accessible format?

Not applicable

Reviewer Expertise:

Behaviour in infection prevention, including sexual health behaviours and STI.

I confirm that I have read this submission and believe that I have an appropriate level of expertise to confirm that it is of an acceptable scientific standard.

# Reviewer report: Reviewer response for version 2

**DOI:** 10.21956/hrbopenres.15748.r52744

**Published:** 2026-01-10

**Authors:** Abhishek Lachyan

**Affiliations:** 1 VMMC and Safdarjung Hospital, New Delhi, Delhi, India

I have carefully reviewed the revised version of the manuscript and the authors’ detailed responses to the reviewers’ comments. The authors have adequately addressed the major points raised in the previous review, particularly by substantially strengthening the *Data Synthesis* section and clearly outlining the planned narrative synthesis approach. The additional clarification regarding multi-level analysis, consideration of the COVID-19 pandemic as a contextual factor, and prioritisation of under-represented populations enhances the methodological transparency and rigor of the protocol.

 Overall, the revisions improve the clarity, coherence, and reproducibility of the proposed review. I have no further substantive comments to make and am satisfied that the manuscript is suitable for indexing in its current form.

Is the study design appropriate for the research question?

Yes

Is the rationale for, and objectives of, the study clearly described?

Yes

Are sufficient details of the methods provided to allow replication by others?

Partly

Are the datasets clearly presented in a useable and accessible format?

Not applicable

Reviewer Expertise:

NA

I confirm that I have read this submission and believe that I have an appropriate level of expertise to confirm that it is of an acceptable scientific standard.

## References

[1] : Predictors of Recent Incidence Trends in Sexually Transmitted Infections: A Systematic Review Protocol. *HRB Open Research* .2026;8: 10.12688/hrbopenres.14222.2 10.12688/hrbopenres.14222.2 PMC1285941041626040

# Reviewer report: Reviewer response for version 1

**DOI:** 10.21956/hrbopenres.15642.r50914

**Published:** 2025-11-27

**Authors:** Chantal den Daas

**Affiliations:** 1 University of Aberdeen, Aberdeen, UK

The presented protocol for a systematic review addresses an important topic. Exploring trends in the most important STI, and looking at predictors of these trends, globally. I think this is an admirable goal, and I would be very interested in the results of this review. Especially if the predictors go beyond standard sociodemographic characteristics and incorporate both individual and societal level constructs.  I think the approach around reviewing, data collection, data protection etc is completely sound. No comments on the approach.

 I do wonder about the nuance, in ‘By examining global epidemiological data, this review aims to provide a nuanced understanding of the multifaceted drivers of increasing STI rates’. I would expect you to find an enormous number of papers, narratively reviewing all predictors might results in a long list, with dependencies on STI, populations, locations etc. I provide some very specific questions (with one country example) on this below. How will depth and breath be offset?

 The timeframe selected includes the Covid-19 pandemic. This pandemic will have had enormous influence on the trends, and the underlying behaviours etc. How will this be accounted for in the review?

 The search strategy includes only English publications. This will limit your findings. For example the type of data that is very relevant to your question in the Netherlands is published in yearly Dutch reports (latest one: https://rivm.openrepository.com/entities/publication/609e455b-6846-41eb-83b3-e7ad82619c57). Reporting trends in STI over time, for all diagnosed cases in municipal health services and GP practices (as far as possible), so not even a sample. Researcher in that institute will publish using this data, but it will always be more specific questions, as the report already does the more basic epidemiology reporting.

 I also wonder how you will incorporate those societal trends that affect STI diagnoses. Again, using the Netherlands as an example, they have recently stopped testing young people for Chlamydia, unless they are presenting with symptoms, this decision will have enormous effects on the epidemiology and trends. You often won’t find these types of national decisions in the publications in your search.  

 HIV is included in STI, small pedantic point, HIV is a virus, might need to state this somewhere in the introduction.

Is the study design appropriate for the research question?

Yes

Is the rationale for, and objectives of, the study clearly described?

Yes

Are sufficient details of the methods provided to allow replication by others?

Yes

Are the datasets clearly presented in a useable and accessible format?

Not applicable

Reviewer Expertise:

Behaviour in infection prevention, including sexual health behaviours and STI.

I confirm that I have read this submission and believe that I have an appropriate level of expertise to confirm that it is of an acceptable scientific standard, however I have significant reservations, as outlined above.

# Response

**Published:** 2025-11-28

**Authors:** Chris Noone

**Affiliations:** School of Psychology, University of Galway, Galway, County Galway, Ireland

We appreciate your time and constructive observations on our protocol paper. We address each of your comments/suggestions as follows.

 1) Our aim, within the scope of the paper, is indeed to summarise available information ideally that “go beyond standard sociodemographic characteristics”. How will we balance depth and breath?

**Response: **This is a very reasonable question, though one that it is difficult to answer a priori. Assuming that we encountered a wide breath of predictors being reported across the studies, we will tabulate these and include them all in an appendix. We will go into detail only for those factors for which there are more than one study, to provide a point of reference/counter-bias. If necessary (i.e., if there is too much data), we will further prioritise detailed observations for minority groups typically under-represented in research (e.g., trans community) and intersections within (e.g., migrant/black/transgender). We have noted this in sections “Outcomes and prioritisation”, and “Data Synthesis”.

 2) How will [Covid-19 pandemic] be accounted for in the review?

**Response: **We agree with the reviewer that this was an important event that affected services and STIs monitoring health systems around the world. As such, we will critically evaluate whether the studies included in our sample controlled for such a factor and contextualise our interpretation of the studies accordingly. This has now been explicitly noted in section “Data Synthesis”.

 3) The search strategy includes only English publications.

**Response: **Yes. English has long been established as “the language of science” enabling the scientific community to communicate and collaborate globally. This not only impact the information that everybody has access to, but it also has practical scientific implications. For example, systematic search strategies are not always directly translatable/replicable across languages. Having said that, although we are searching records based on concepts in English, we will make an exception for studies published also in Spanish and Italian, since these are languages spoken within the research team (this was explicitly stated under the “Exclusion criteria”, bullet point 2). Although ideal, to our knowledge, very few research teams will have the resources to undertake an international literature review in native languages around the globe. 

 4) How you will incorporate those societal trends that affect STI diagnoses. Again, using the Netherlands as an example, they have recently stopped testing young people for Chlamydia, unless they are presenting with symptoms, this decision will have enormous effects on the epidemiology and trends. You often won’t find these types of national decisions in the publications in your search. 

**Response: **This is a really good point. However, methodologically—and realistically—, we can only include and review the information provided in the source papers/records. As the study is framed within a critical realist perspective, we will attempt to incorporate societal factors that are reported (e.g., via tabulation/vote count) using a multi-level approach (i.e., identifying factors at the individual, interpersonal, community, institutional/health system, structural). As scientists, if we were reporting epidemiological data from a country of which we know a significant structural change affected data trends from a particular point in time, we would include and report such a factor—we can only expect the same from other professionals. We do try to address your concern through our grey literature search which includes policy documents.

 5) HIV is included in STI,… [but it] is a virus, might need to state this somewhere in the introduction.

**Response: **We disagree with the suggestion. Current terminological preference favours talking about “infections” (as opposed to “diseases”/STDs) as it is deemed to be less stigmatizing. To the point, however, the Cambridge dictionary’s definition of infection is “a condition in which bacteria or viruses that cause disease have entered the body”. Other standard definitions within the biological sciences include the generic term “microorganisms” of which bacteria and viruses alike are included as conveyers/causes of diseases. We argue it is correct to include HIV within the STI umbrella term.

# Reviewer report: Reviewer response for version 1

**DOI:** 10.21956/hrbopenres.15642.r50677

**Published:** 2025-10-14

**Authors:** Abhishek Lachyan

**Affiliations:** 1 VMMC and Safdarjung Hospital, New Delhi, Delhi, India

This manuscript presents a systematic review protocol aiming to identify predictors of recent trends in four priority sexually transmitted infections (STIs): gonorrhoea, chlamydia, syphilis, and HIV. The authors justify the study by highlighting the increasing incidence of these STIs globally and the significant public health burden they pose. The review will focus on longitudinal data published between 2015 and 2025, aiming to capture structural, behavioural, biological, and systemic factors that influence incidence trends.

 The protocol outlines a comprehensive search strategy across multiple databases and grey literature, clear inclusion and exclusion criteria, and a screening process with calibration exercises to ensure reliability. Data extraction and narrative synthesis will follow established guidelines, with risk of bias assessed using validated tools. The authors also highlight their commitment to transparency and data sharing, indicating that the datasets and code will be made available via OSF.

 The manuscript is methodologically sound and relevant, with clear rationale and objectives. However, certain aspects—such as providing full database-specific search strings, a sample data extraction template, and more detail on the narrative synthesis approach—require clarification to ensure full replicability and transparency. Addressing these points would strengthen the manuscript and make it fully robust.

Is the study design appropriate for the research question?

Yes

Is the rationale for, and objectives of, the study clearly described?

Yes

Are sufficient details of the methods provided to allow replication by others?

Partly

Are the datasets clearly presented in a useable and accessible format?

Not applicable

Reviewer Expertise:

NA

I confirm that I have read this submission and believe that I have an appropriate level of expertise to confirm that it is of an acceptable scientific standard, however I have significant reservations, as outlined above.

# Response

**Published:** 2025-11-28

**Authors:** Chris Noone

**Affiliations:** School of Psychology, University of Galway, Galway, County Galway, Ireland

We are grateful for your positive review of our protocol paper. We have revised it in response to your comment below:

 1) "… providing full database-specific search strings, a sample data extraction template, and more detail on the narrative synthesis approach—require clarification to ensure full replicability and transparency."

 We couldn’t agree more. We had generated a search string for each database following Bramer et al.’s (2018) guidelines on search standardisation. Such information was made available via the project’s OSF site ( https://osf.io/48m9p already referenced in the protocol), specifically: https://osf.io/g5mds

 We have also elaborated on the narrative synthesis approach.
